# Numerical Study of a Dual-Mode Optical Sensor for Temperature and Refractive Index Sensing with Enhanced Temperature Range

**DOI:** 10.3390/s25133999

**Published:** 2025-06-26

**Authors:** Muhammad Favad Qadir, Muhammad Zakwan, Saleem Shahid, Ahsan Sarwar Rana, Muhammad Mahmood Ali, Wolfgang Bösch

**Affiliations:** 1 Department of Electrical and Computer Engineering, Air University, Islamabad 44230, Pakistan; favad.qadir@au.edu.pk (M.F.Q.); ahsan.sarwar@au.edu.pk (A.S.R.); 2 Faculty of Engineering and Computing, Department of Electrical Engineering, National University of Modern Languages, Islamabad 44000, Pakistan; 3 Institute of Avionics and Aeronautics, Air University, Islamabad 44230, Pakistan; mzakwan@au.edu.pk; 4 Institute of Microwave and Photonic Engineering, Graz University of Technology, 8010 Graz, Austria; wbosch@tugraz.at; 5 Department of Mechatronic Engineering, Atlantic Technological University, F91 YW50 Sligo, Ireland; muhammad.ali@atu.ie

**Keywords:** SOI waveguide, integrated optical sensors, dual polarization, simultaneous measurement

## Abstract

This study presents a photonic integrated optical sensor based on a dual-polarization microring resonator with angular gratings on a silicon-on-insulator (SOI) waveguide, enabling simultaneous and precise refractive index (RI) and temperature measurements. Due to the distinct energy distributions for transverse electric (TE) and transverse magnetic (TM) modes in SOI waveguides, these modes show distinct sensitivity responses to the variation in ambient RI and temperature. Simultaneous measurements of both temperature and RI are enabled by exciting both these transverse modes in the microring resonator structure. Furthermore, incorporating angular gratings into the microring resonator’s inner sidewall extends the temperature measurement range by mitigating free spectral range limitations. This work presents a novel approach to dual-polarization microring resonators with angular gratings, offering an enhanced temperature measurement range and detection limit in optical sensing applications requiring an extended temperature range. The proposed structure is able to yield a simulated temperature measurement range of approximately 35 nm with a detection limit as low as $2.99\times 10^{-5}$. The achieved temperature sensitivity is 334 pm/°C and RI sensitivity is 13.33 nm/RIU for the ${\mathrm{TE}}_{0}$ mode, while the ${\mathrm{TM}}_{0}$ mode exhibits a temperature sensitivity of 260 pm/°C and an RI sensitivity of 76.66 nm/RIU.

## 1. Introduction

In optical sensing, the simultaneous measurement of multiple parameters is a significant area of ongoing research, with numerous structures based on fiber optics and integrated optics employed to achieve the accurate detection of these parameters. Temperature and RI are among the most critical parameters due to their interdependence, necessitating simultaneous and precise measurements. Various structures, including optical fibers [1,2,3,4,5] and planar waveguides [6], have been utilized for this purpose. Recent advancements have introduced complex configurations, such as those involving artificial neural networks [7,8], plasmon resonance [9], interferometers [10,11], the Fabry–Perot cavity, [12,13,14], fiber SPR [15], and photonic crystal-based nanobeam cavities [16,17]. However, optical microcavities like microring resonators (MRRs) stand out due to their simplicity and robustness. In MRRs, the resonating wavelength shifts in response to the variations in ambient temperature and RI; however, this shift is constrained by the free spectral range (FSR) [18], limiting the simultaneous sensing range for RI and temperature.

Expanding the sensing range is crucial for comprehensive environmental monitoring and industrial applications, where the accurate detection of diverse and dynamic changes in RI and temperature is essential. Given that these parameters are often interdependent, their variations can significantly impact system performance and safety. A broader sensing range allows integrated optical sensors to provide more detailed and reliable data, enhancing decision-making processes and operational efficiency. Although numerous studies have explored methods to extend the measurement range of MRR-based sensors by incorporating angular gratings [19], cascaded ring resonators [20], and variations in the effective group index [21], there has been little focus on improving the accuracy of these sensors under varying temperature conditions [1]. However, by simultaneously measuring the RI and temperature in an MRR sensor, the accuracy of these parameters can be improved.

This study presents a dual-polarization angular-grating microring resonator (DP AG-MRR) designed for the precise detection of both RI and temperature over an extended measurement range. Unlike the dual-resonance approach utilized in metal-clad ridge waveguide (MCRW) structures, which is primarily focused on RI sensing [22], the proposed DP AG-MRR employs a simplified silicon-on-insulator (SOI) platform capable of simultaneously measuring both RI and temperature. The dual-mode excitation of ${\mathrm{TE}}_{0}$ and ${\mathrm{TM}}_{0}$ is made possible through a directional coupler-like bend access waveguide, whereas the extended temperature measurement capability is enabled by embedding angular gratings in the sidewalls of the MRR, which predominantly induce the Bragg effect for the ${\mathrm{TE}}_{0}$ mode. For simplicity of design in the coupling section, a directional coupler-like bend waveguide is employed instead of more complex asymmetric coupling designs. The effectiveness of the designed DP AG-MRR sensor is evaluated using the 2.5D VarFDTD method. The sensor’s response to temperature and RI changes is characterized by the differential energy distributions of the fundamental ${\mathrm{TE}}_{0}$ and ${\mathrm{TM}}_{0}$ modes in the SOI waveguide, resulting in distinct spectral peaks for each polarization.

## 2. Materials and Methods

### 2.1. Structure Design and Working Principle

**Figure 1:**
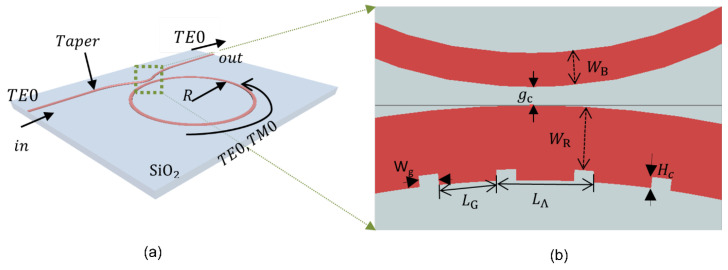
(**a**) Structural layout of the proposed DP AG-MRR. (**b**) Upper view taken at the center of the coupling section.

To construct the optical sensor, an SOI-based photonic platform was utilized, featuring a silicon device layer with a thickness of 220 nm and an insulator layer of 2 µm, topped with air cladding. The refractive indices of the silicon device, insulator layer, and air cladding were 3.48, 1.44, and 1.0, respectively. Figure 1a illustrates a schematic of the proposed structure.

In the design of the optical sensor, the input bus waveguide width was set at 500 nm to facilitate single-mode ${\mathrm{TE}}_{0}$ propagation. Near the coupling section, a narrower bus waveguide width $W_{B}$ of approximately 295 nm was employed to enhance coupling efficiency with the ring waveguide. An adiabatic taper section bridges the bus waveguides at the input and the coupling sections. The ring waveguide itself was approximately 550 nm wide, supporting efficient propagation of both ${\mathrm{TE}}_{0}$ and ${\mathrm{TM}}_{0}$ modes. It features a bend radius of 10 µm, with reference to the circle’s center of the ring waveguide. At the coupling section, the gap between the ring and bus waveguides $g_{c}$ was approximately 0.16 µm. The angular grating dimensions with length $L_{G}$, height $H_{c}$, and grating gap $W_{g}$ were 0.568 µm, 0.045 µm, and 0.042 µm, respectively, with a grating period $\left(L_{\Lambda }\right)$ of about 0.61 µm, as depicted in Figure 1b. The waveguide sections with widths $W_{R}$ + $H_{c}$ and $W_{R}$ were 595 nm and 550 nm, respectively. The duty cycle D of the grating was selected at approximately 93%. The overall device dimensions were less than 42μm×25μm.

**Figure 2:**
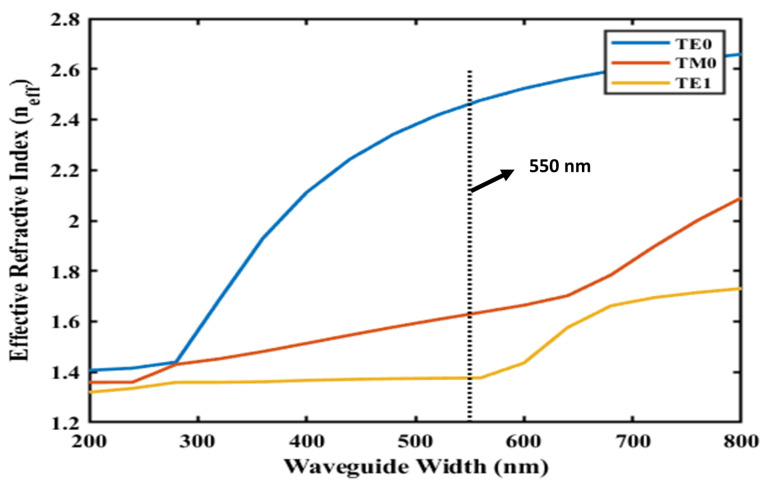
Dependence of the effective refractive index $\left(n_{\mathrm{eff}}\right)$ on waveguide width for air cladding.

The operating principle of the microring resonator is predicated on the simultaneous excitation of the ${\mathrm{TE}}_{0}$ and ${\mathrm{TM}}_{0}$ modes within the microring cavity. This can be achieved by exciting a continuous-wave broadband light source. The input waveguide, with a thickness of 500 nm, supports only the ${\mathrm{TE}}_{0}$ mode, after which the light enters the taper section and couples with the ring waveguide. The width of the ring waveguide was set to 550 nm, enabling the simultaneous support of both ${\mathrm{TE}}_{0}$ and ${\mathrm{TM}}_{0}$ modes, as shown in Figure 2. Furthermore, it was constructed to accommodate fabrication imperfections. Even with usual deviations during fabrication [23], the mode coupling efficiency was maintained, and both ${\mathrm{TE}}_{0}$ and ${\mathrm{TM}}_{0}$ modes could be excited and propagated without significant degradation. To mitigate alignment sensitivities, a taper was employed to ensure a gradual transition for stable coupling into the microring waveguide. The taper’s position relative to the ring waveguide was also optimized to facilitate the efficient coupling of both ${\mathrm{TE}}_{0}$ and ${\mathrm{TM}}_{0}$ modes.

To simplify the phase-matching conditions in the coupling section, a directional coupler-like structure was employed, featuring a zero coupling length and a coupling gap of approximately 0.16 µm. For the optical microring resonator, the resonance condition is described as follows:

(1) $${m\lambda }_{\mathrm{res}}=n_{\mathrm{eff}}\cdot L$$

where m denotes the azimuthal mode number associated with the longitudinal resonances (m = 1, 2, 3, …), $\lambda_{\mathrm{res}}$ represents the resonating wavelength, and $n_{\mathrm{eff}}\cdot L$ signifies the effective optical length of the microring resonator. To achieve this dual mode propagation, the width of the ring waveguide was set at 550 nm when the cladding was air, as shown in Figure 2. This shows that ${\mathrm{TE}}_{1}$ modes also propagate in ring waveguides, but they exhibit poor mode confinement within the ring waveguide and tend to diminish over time. Consequently, only the ${\mathrm{TE}}_{0}$ and ${\mathrm{TM}}_{0}$ modes persisted, as illustrated in Figure 1a.

**Figure 3:**
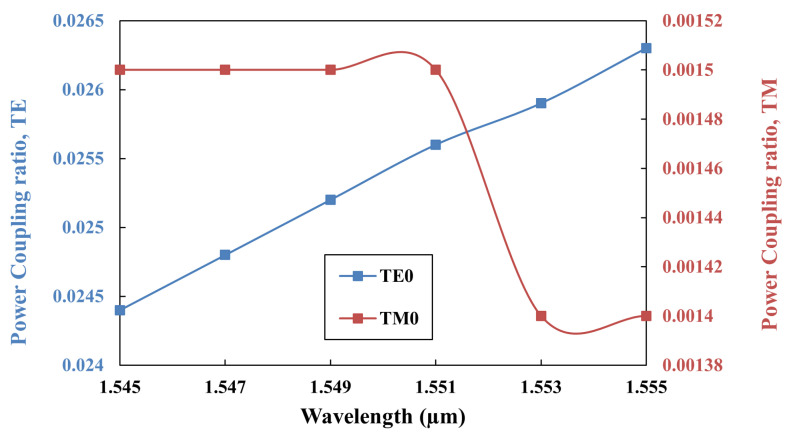
Power coupling ratio from bus waveguide mode to the ring mode.

The coupling strength between the fundamental ${\mathrm{TE}}_{0}$ mode of the bus waveguide and that of the ring waveguide was notably higher compared to that between the ${\mathrm{TE}}_{0}$ mode of the bus waveguide and ${\mathrm{TM}}_{0}$ mode of the ring waveguide. This discrepancy arises because coupling between identical polarization modes is inherently more efficient than between different polarization modes. Figure 3 displays the power coupling ratio of the ${\mathrm{TE}}_{0}$ mode in the bus waveguide to both the ${\mathrm{TE}}_{0}$ and ${\mathrm{TM}}_{0}$ modes supported by the ring waveguide and highlights the distinct extinction ratios attributable to their differing power coupling efficiencies.

**Figure 4:**
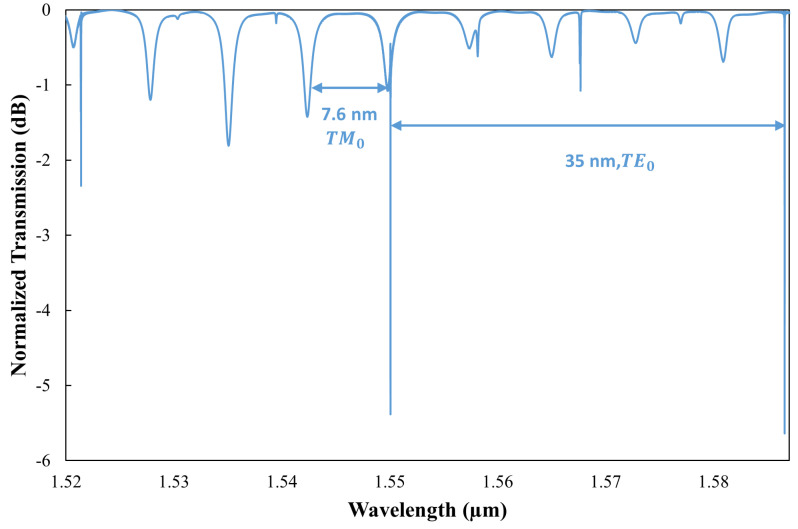
Simulated normalized transmission spectrum of DP AG-MRR.

For sensing applications, where shifts in the resonance wavelength are critical, the sensing range is governed by the free spectral range (FSR), which corresponds to the spectral spacing between successive longitudinal modes of the ring resonator. However, this range is subject to certain limitations, as shown below:

(2) $$\delta \lambda_{\mathrm{res}}<\mathrm{FSR}$$

where $\delta \lambda_{\mathrm{res}}$ indicates the variation in the resonance wavelength. To enhance the measurement range, this shift must be greater than the FSR between the longitudinal modes. To achieve this shift, the incorporation of angular gratings on the inner sidewall of the microring resonator has been proposed. This modification enables selective wavelength operation independent of the FSR and hence facilitates the measurement of target parameters over an expanded range. Our target is to achieve the dominant mode that is able to measure a larger temperature range while measuring RI simultaneously. So, we designed the angular gratings on the inner wall of the microring resonator in such a way that they will facilitate Bragg reflection predominantly for the ${\mathrm{TE}}_{0}$ mode and not for the ${\mathrm{TM}}_{0}$ mode. Consequently, the device’s response selectively filters out side modes surrounding the dominant mode for ${\mathrm{TE}}_{0}$ polarization, leading to a more pronounced resonance for the ${\mathrm{TE}}_{0}$ mode relative to the ${\mathrm{TM}}_{0}$ mode, as shown in Figure 4. This figure illustrates the simulated normalized power transmission of the dual-polarization angular-grating microring resonator (DP AG-MRR) when the cladding is air. The transmission spectrum is quantified as the ratio of the output-to-input power integrals. The observed variations in the extinction ratios for the ${\mathrm{TE}}_{0}$ and ${\mathrm{TM}}_{0}$ modes are attributed to their distinct power coupling ratios, as discussed previously. The FSR for the ${\mathrm{TE}}_{0}$ mode is extended to approximately 35 nm due to the suppression of side modes. The FSR for the ${\mathrm{TM}}_{0}$ mode is approximately 7.6 nm, displaying no dominant mode.

**Figure 5:**
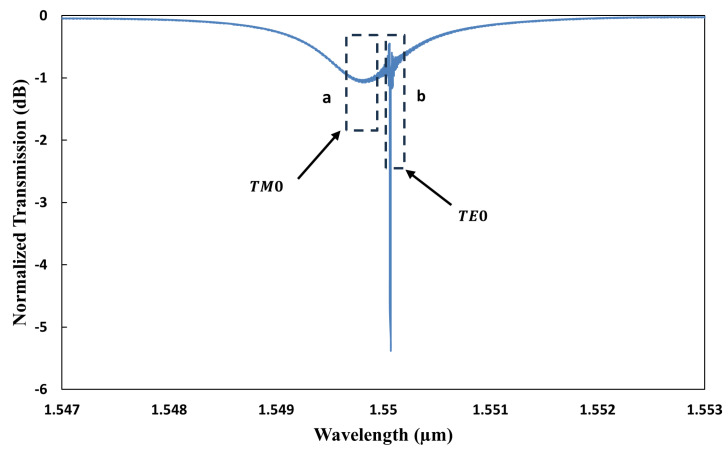
(**a**) ${\mathrm{TM}}_{0}$. (**b**) ${\mathrm{TE}}_{0}$.

**Figure 6:**
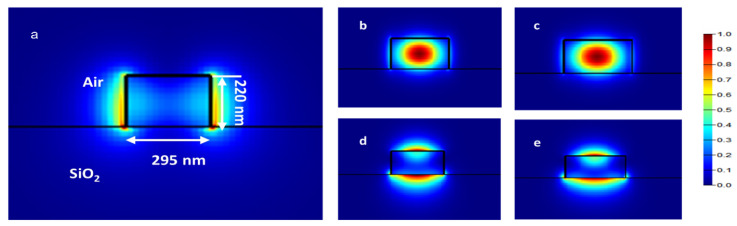
(**a**) Normalized electric field distribution at input bus waveguide near coupling section. (**b**) Quasi TE01 fundamental mode profile at wavelength 1550 nm with waveguide of 550 nm and (**c**) 595 nm. (**d**) Quasi TM01 mode profile with waveguide width of 550 nm. (**e**) TM01 mode profile with waveguide width of 595 nm.

Figure 5 shows dual resonances visible in the output transmission spectrum corresponding to the ${\mathrm{TE}}_{0}$ and ${\mathrm{TM}}_{0}$ modes. Figure 6a illustrates the normalized electric field profile of the ${\mathrm{TE}}_{0}$ mode in the input waveguide, positioned near the coupling region. Additionally, the mode profiles for the ${\mathrm{TE}}_{0}$ and ${\mathrm{TM}}_{0}$ polarizations, corresponding to the relative widths of the waveguides, are depicted in Figure 6b–e.

The proposed dual-polarization microring resonator with angular gratings (DP AG-MRR) could be fabricated on a 220 nm thick silicon-on-insulator wafer by following a standard fabrication process for silicon photonic devices. The process would begin with spin-coating a 250–300 nm thick electron beam photoresist layer and then defining the microring resonator and waveguide structures using electron beam lithography. This patterned layout is subsequently transferred onto the silicon device layer using inductively coupled plasma reactive ion etching (ICP-RIE), using an C4F8 and SF6 gas mixture [24,25]. Given that manufacturing tolerances can impact the performance of conventional microring resonators [23], careful process calibration would be essential. Lastly, grating couplers could be incorporated at the input/output waveguide facets using an overlay exposure and a shallow etch to facilitate efficient fiber-to-chip coupling [26]. A potential approach for experimentally evaluating the proposed device is to couple light from a tunable laser or a continuous-wave (CW) broadband source, typically operating near 1550 nm, into the chip via polarization-sensitive grating couplers. The chip’s output transmission spectrum can then be recorded using an optical spectrum analyzer [27].

### 2.2. Parametric Analysis

The device’s performance was optimized through the application of the 2.5-dimensional variational finite-difference time-domain (var-FDTD) approach. For broadband simulations, the variational effective index method was employed. Mesh accuracy was maintained at a value of 4, with a minimum mesh step size of 0.00025 µm selected to effectively balance precision, memory requirements, and simulation duration. The mesh resolution is defined by a minimum step size of 250 pm. In the x, y, and z dimensions, perfectly matched layer (PML) boundaries were employed to absorb incident light with minimal reflection. To further enhance simulation robustness, a stabilized PML profile along the x-axis was implemented, supplemented with additional layers. This configuration utilizes a stretched coordinate PML, improving absorption efficiency and reducing reflection at the boundaries.

The dual-polarization angular-grating microring resonator (DP AG-MRR) was evaluated by injecting light from a fundamental TE-mode source into the input port of the bus waveguide, as described in [28]. Structural parameters, including $g_{c}$ = 160 nm, $H_{c}$ = 40 nm, and D=93%, were meticulously optimized. The device demonstrated a pronounced resonant peak for the ${\mathrm{TE}}_{0}$ mode at the operational wavelength of 1550 nm, within a spectral range of 1500–1600 nm. The conventional ring resonator featured a free spectral range (FSR) of approximately 9.5 nm, whereas the FSR for the dominant and secondary peaks of the DP AG-MRR was about 35 nm, nearly threefold that of the conventional design for the ${\mathrm{TE}}_{0}$ mode.

### 2.3. Quality Factor, Extinction Ratio, and Side-Mode Suppression

The extinction ratio (ER) and quality factor are critical metrics for assessing the detection capabilities of optical sensors, particularly in terms of sensitivity and detection limits. To attain enhanced sensitivity and minimal detection limits, it is imperative to rigorously evaluate the ER and *QF* of the proposed sensor. The quality factor is defined as

(3) $$Q=\frac{\lambda }{\Delta \lambda }$$

where λ denotes the resonant wavelength, and Δλ represents the full width at half maximum (FWHM) of the resonance. To achieve a superior quality factor (QF), it is crucial to minimize losses, which in the DP AG-MRR primarily arise from propagation and coupling. The coupling length is effectively set to zero by opting for a ring-shaped rather than racetrack-shaped resonator, as demonstrated in [29], thereby reducing losses to a minimal extent. The scattering losses due to angular gratings are below 13% [30]. The excess bending losses for a 550 nm wide ring waveguide are less than 0.009 dB/90° [31] for a bend radius of 10 µm, which accounts for mode mismatch losses. A bent waveguide with a width of 295 nm was utilized to optimize coupling, with a coupling gap $g_{c}$ of approximately 160 nm. Other parameters such as the *D* and $H_{c}$ were also carefully selected at 93% and 40 nm, respectively.

**Figure 7:**
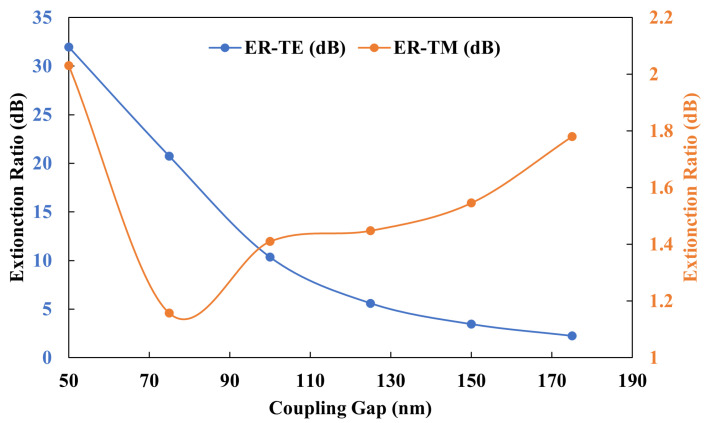
Extinction ratio and coupling gap ($g_{c}$).

The dependence of the extinction ratio on the coupling gap $g_{c}$ is depicted in Figure 7. Within this figure, it is evident that the ER for the ${\mathrm{TE}}_{0}$ mode decreases as $g_{c}$ increases. Conversely, the extinction ratio for the ${\mathrm{TM}}_{0}$ mode exhibits a more gradual variation. A $g_{c}$ of 160 nm was strategically selected to facilitate clear differentiation between the extinction ratios of the ${\mathrm{TE}}_{0}$ and ${\mathrm{TM}}_{0}$ modes.

**Figure 8:**
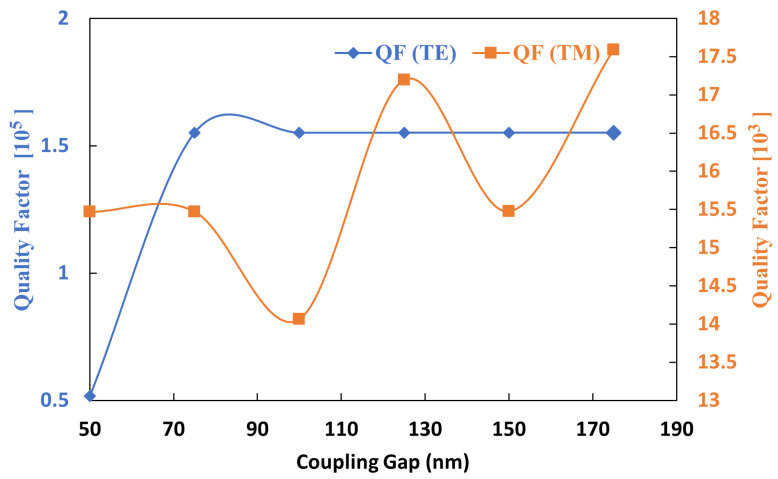
Quality factor and coupling gap ($g_{c}$).

The relationship between quality factor QF and coupling gap $g_{c}$ is illustrated in Figure 8. The QF for the ${\mathrm{TM}}_{0}$ mode exhibits minimal variation and remains nearly constant, while QF for the ${\mathrm{TE}}_{0}$ mode shows a rapid increase at lower $g_{c}$ values before stabilizing at higher values. A $g_{c}$ of approximately 160 nm was selected to optimize the QF, achieving an elevated level of approximately $1.55\times 10^{5}$, which is particularly advantageous for targeted ${\mathrm{TE}}_{0}$ mode temperature sensing.

**Figure 9:**
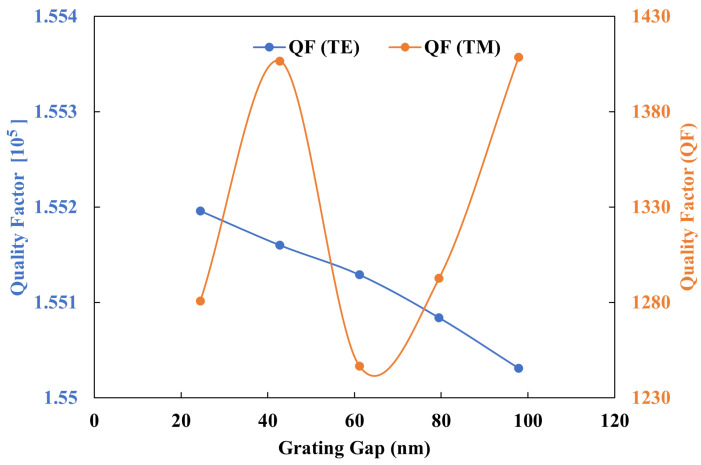
Quality factor as a function of the grating gap for the ${\mathrm{TE}}_{0}$ and ${\mathrm{TM}}_{0}$ modes.

The duty cycle is a key design parameter in the development of the dual-polarization angular-grating microring resonator (DP AG-MRR). The higher duty cycle generally increases the grating strength, which in turn enhances coupling efficiency and side-mode suppression. However, excessively high duty cycles may result in increased coupling between TE and TM modes, consequently reducing side-mode suppression. Figure 9 illustrates the relationship between the QF and $W_{g}$ for the transverse electric and transverse magnetic modes. It is observed that an increase in the duty cycle corresponds to an increase in QF, albeit with a concomitant decrease in the grating gap $W_{g}$. Given the complexity of fabricating smaller grating heights, a trade-off between QF and the grating gap is necessary. Consequently, a duty cycle of 93% with a grating height $H_{c}$ of about 45 nm was selected. The duty cycle for the ${\mathrm{TM}}_{0}$ mode displays a non-linear relationship and is not the focus of this study. However, it is noteworthy that the maximum QF for the ${\mathrm{TM}}_{0}$ mode occurs at a 93% duty cycle.

**Figure 10:**
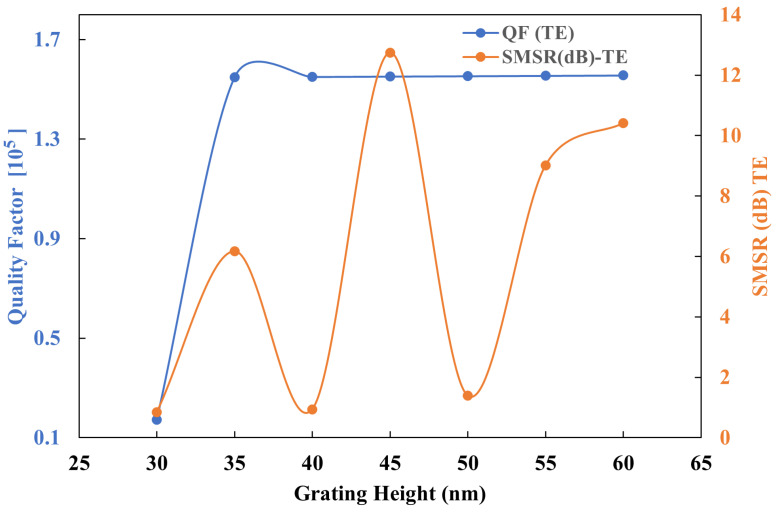
QF and SMSR as a function of grating height for ${\mathrm{TE}}_{0}$ mode.

The influence of grating height on the QF and side-mode suppression ratio (SMSR) for the ${\mathrm{TE}}_{0}$ mode was evaluated by varying the grating height from 30 nm to 60 nm. As illustrated in Figure 10, the optimal SMSR is achieved at a grating height of 45 nm, which is critical for distinguishing between the ${\mathrm{TE}}_{0}$ and ${\mathrm{TM}}_{0}$ modes. Notably, the QF remains relatively constant for the ${\mathrm{TM}}_{0}$ mode throughout this range. $g_{c}$ and D are maintained at 160 nm and 93%, respectively.

**Figure 11:**
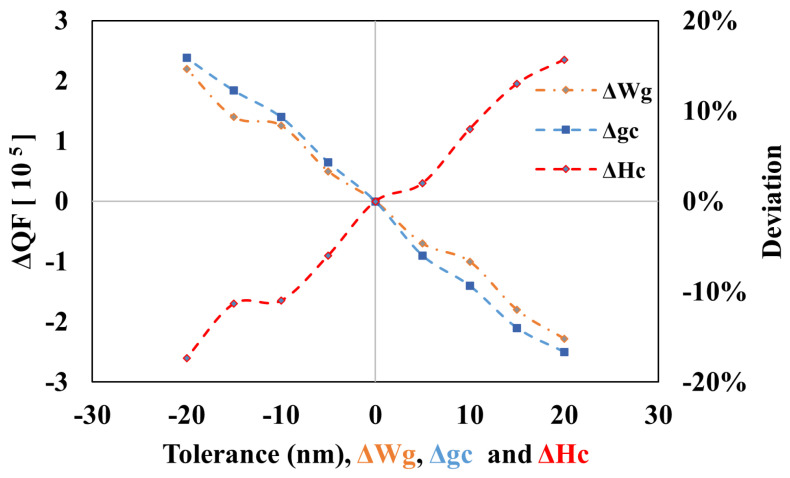
Deviation in QF from the optimal value.

**Figure 12:**
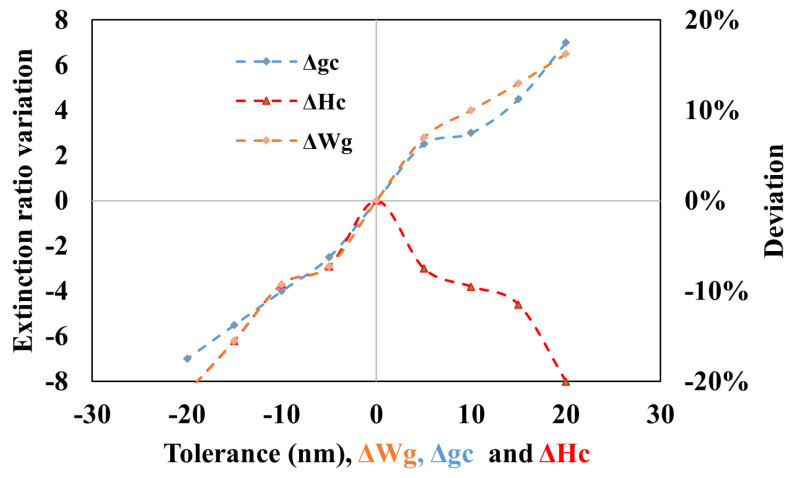
Impact of dimension variations in DP AG-MRR for extinction ratio.

The effect of fabrication tolerance [23] on the the features of traditional DP AG-MRR is presented in Figure 11 and Figure 12. For the proposed device, the effects associated with the angular grating parameters such as $W_{g}$, $H_{c}$, and coupling gap $g_{c}$ are discussed. The optimal device parameters, corresponding to a D of 93%, include a $W_{g}$ of 0.042 µm, $H_{c}$ of 0.045 µm, and $g_{c}$ of 0.16 µm. With these parameters, the QF is $1.55\times 10^{5}$, and the ER is 3 dB for the target ${\mathrm{TE}}_{0}$ mode. Figure 11 and Figure 12 illustrate the effects of changes in $W_{g}$, $g_{c}$, and $H_{c}$ on the deviations QF and ER from their optimal values. From Figure 11, it is observed that the variation in QF and ER is less than 20% for the change in $W_{g}$ and $g_{c}$. The deviation in QF and ER is consistent for these variations. However, as the tolerance of $\Delta H_{c}$ increases, the deviation in ΔQF also increases, exhibiting opposite behavior for lower tolerance values. The variation in ER from lower to higher tolerance in $\Delta H_{c}$ is inconsistent, which can be attributed to the dual-mode overlapping behavior of ${\mathrm{TE}}_{0}$ and ${\mathrm{TM}}_{0}$ modes. To achieve better results and minimize fabrication tolerance, advanced fabrication technologies with precision on the scale of a few nanometers are required.

## 3. Results and Discussion

### 3.1. Resonant Wavelength Shift

**Figure 13:**
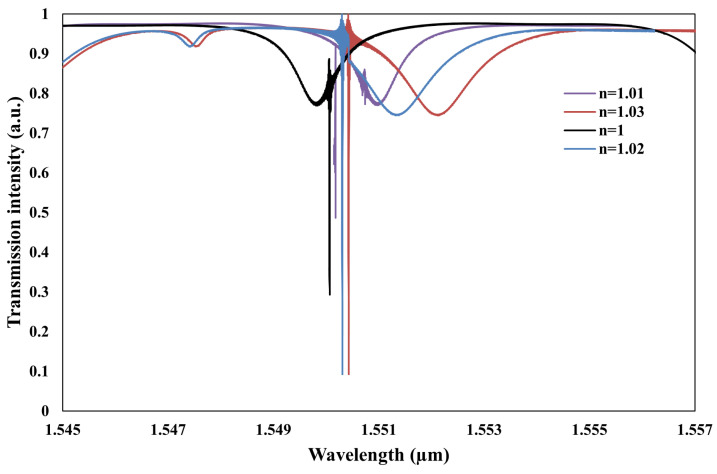
Transmission intensity of the proposed structure for range of air (1) to chemical gases (n = 1 to 1.03).

Figure 13 presents the simulated transmission spectra for environments of air (n = 1) and chemical gases (n = 1 to 1.03). The analysis indicates a resonance wavelength shift of 2.4 nm for the ${\mathrm{TM}}_{0}$ mode, which is limited by the FSR and is not the primary focus of this study. In contrast, a smaller shift of 0.36 nm is observed for the ${\mathrm{TE}}_{0}$ mode. Notably, the ${\mathrm{TM}}_{0}$ mode exhibits greater light confinement within the cladding region compared to the waveguide region. As depicted in Figure 6, the sensitivities for different polarization types are quantified as $S_{\mathrm{RI},\;{\mathrm{TM}}_{0}}=76.66\;\mathrm{nm}/\mathrm{RIU}$ and $S_{\mathrm{RI},\;{\mathrm{TE}}_{0}}=13.33\;\mathrm{nm}/\mathrm{RIU}$.

**Figure 14:**
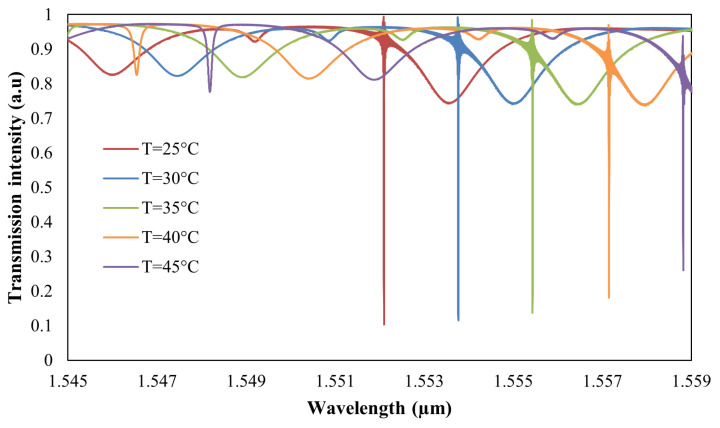
Transmission response of the proposed structure under varying ambient temperatures.

Figure 14 presents the variation in the resonance wavelength of the ${\mathrm{TE}}_{0}$ mode with respect to temperature, observed with air cladding. The spectral shift of the resonance wavelength corresponding to the ${\mathrm{TE}}_{0}$ is recorded at 1.67 nm, which is greater than the 1.30 nm shift observed for the ${\mathrm{TM}}_{0}$ mode. This variation is due to the significantly higher thermo-optic coefficient of silicon $1.8\times 10^{-4}\times K^{-1}$ [17], in contrast to that of air cladding $9.8\times 10^{-7}\times K^{-1}$ [32]. Consequently, the temperature sensitivity of the ${\mathrm{TE}}_{0}$ mode, quantified at $S_{T,\;{\mathrm{TE}}_{0}}=334\;\mathrm{pm}/{}^{\circ}C$ exceeds that of the ${\mathrm{TM}}_{0}$ mode, which is measured at $S_{T,\;{\mathrm{TM}}_{0}}=260\;\mathrm{pm}/{}^{\circ}C$. Furthermore, the sensitivity ratios for the RI, $\frac{S_{\mathrm{RI},\;{\mathrm{TM}}_{0}}}{S_{\mathrm{RI},\;{\mathrm{TE}}_{0}}}=5.75$ and temperature $\frac{S_{T,\;{\mathrm{TM}}_{0}}}{S_{T,\;{\mathrm{TE}}_{0}}}=0.78$ differ significantly, facilitating the simultaneous detection of both parameters. Notably, the RI sensitivity is relatively low, indicating that the dual-resonance approach is well-suited for applications where temperature measurement is of primary importance. To enhance RI sensitivity while maintaining temperature sensing capabilities, a viable approach is to incorporate a dual-polarization, subwavelength-based structure. This method will be explored and presented in our future work.

### 3.2. Wavelength Selection

**Figure 15:**
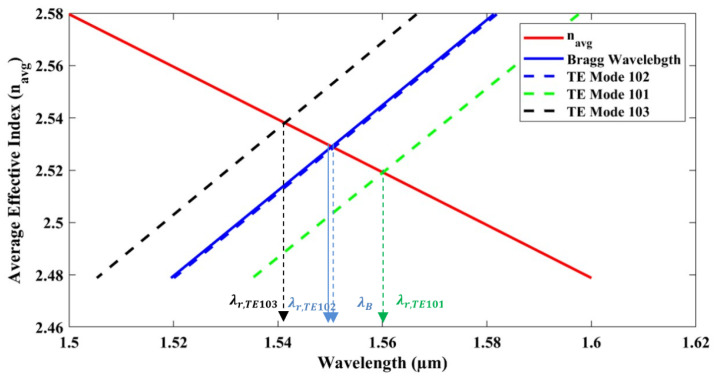
Dominant ${\mathrm{TE}}_{0}$ mode selection of DP AG-MRR.

Wavelength selectivity for the fundamental ${\mathrm{TE}}_{0}$ mode was investigated using eigensolver analysis [33]. This analysis was applied to waveguide sections with widths $W_{R}$ + $H_{c}$ and $W_{R}$, with widths of 595 nm and 550 nm, respectively, as depicted in Figure 1b. To demonstrate wavelength selectivity, Figure 15 features a solid red line representing the equivalent wavelength of the dual-polarization angular-grating microring resonator (DP AG-MRR), calculated using Rytov’s formula [34], as below:

(4) $$n_{\mathrm{avg}}^{2}=D\cdot n_{\mathrm{eff}1}^{2}+\left(1-D\right)\cdot n_{\mathrm{eff}2}^{2}$$

**Figure 16:**
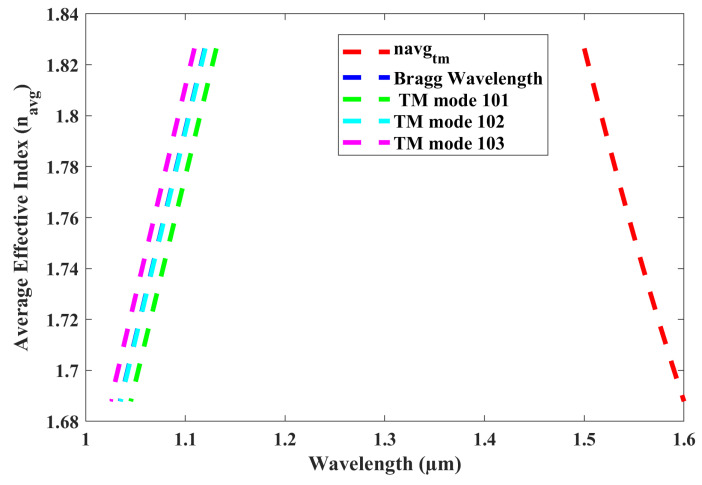
Non-selection of dominant ${\mathrm{TM}}_{0}$ mode in DP AG-MRR.

Here, $n_{\mathrm{eff}1}$ and $n_{\mathrm{eff}2}$ represent the effective refractive indices of the waveguide segments with widths $W_{R}$ + $H_{c}$ and $W_{R}$, respectively. The solid blue line in the diagram represents the Bragg wavelength, intersecting the red line at 1550 nm. The dashed black, blue, and green lines correspond to mode numbers 103, 102, and 101, respectively. The wavelengths for modes 101 and 103 are significantly divergent from the Bragg wavelengths; therefore, only the mode proximate to the Bragg wavelength, specifically mode number 102 (dashed blue line), will be selected as the dominant mode. The wavelength selectivity of the ${\mathrm{TM}}_{0}$ mode will be explored in our forthcoming research, which will include investigations into the dual-polarization angular-grating microring resonator (DP-AG MRR). Figure 16 demonstrates that angular gratings have no impact on the ${\mathrm{TM}}_{0}$ mode, resulting in no wavelength selection from the ${\mathrm{TM}}_{0}$ mode. The dashed red line represents the average effective index of the waveguide, which corresponds to the same wavelength range as the ${\mathrm{TE}}_{0}$ mode, as both modes propagate within the same ring waveguide. The dashed blue line in Figure 16 indicates the Bragg wavelength within the 1.03 to 1.12 µm range, which is significantly different from the 1.5 to 1.6 µm wavelength range. Although mode 102 is close to the Bragg wavelength for the ${\mathrm{TM}}_{0}$ mode, it is not selected because it does not intersect with the dashed red line representing the average effective index of the waveguide. This behavior is evident in the transmission spectrum shown in Figure 4. Notably, this study marks the first successful demonstration of a dominant ${\mathrm{TE}}_{0}$ mode using angular gratings within a dual-polarization microring resonator. Table 1 provides a comparative analysis of the current study on the simultaneous detection of RI and temperature through MRR-based optical sensing.

### 3.3. Sensing Analysis

The sensing characteristics of DP AG-MRR are quantified using the sensitivity matrix ${\mathrm{SM}}_{T,n}$ defined below:

(5) $${\mathrm{SM}}_{T,\mathrm{RI}}=\left[\begin{array}{cc} S_{T,{\mathrm{TE}}_{0}} & S_{\mathrm{RI},{\mathrm{TE}}_{0}}\\ S_{T,{\mathrm{TM}}_{0}} & S_{\mathrm{RI},{\mathrm{TM}}_{0}} \end{array}\right]$$

where $S_{T,{\mathrm{TM}}_{0}}$ and $S_{RI,{\mathrm{TM}}_{0}}$ denote the sensitivities to temperature and RI, respectively, under ${\mathrm{TM}}_{0}$ polarization, while $S_{T,{\mathrm{TE}}_{0}}$ and $S_{RI,{\mathrm{TE}}_{0}}$ represent the corresponding sensitivities under ${\mathrm{TE}}_{0}$ polarization. The variations in RI and temperature can be determined using the following equations:

(6) $$\left[\begin{array}{c} \Delta T\\ \Delta n \end{array}\right]={\mathrm{SM}}_{T,\mathrm{RI}}^{-1}\cdot \left[\begin{array}{c} \Delta \lambda_{{\mathrm{TE}}_{0}}\\ \Delta \lambda_{{\mathrm{TM}}_{0}} \end{array}\right]$$

(7) $$\Delta T=\frac{S_{\mathrm{RI},{\mathrm{TM}}_{0}}\cdot \Delta \lambda_{{\mathrm{TE}}_{0}}-S_{\mathrm{RI},{\mathrm{TE}}_{0}}\cdot \Delta \lambda_{{\mathrm{TM}}_{0}}}{\det({\mathrm{SM}}_{T,\mathrm{RI}})}$$

(8) $$\Delta n=\frac{S_{T,{\mathrm{TE}}_{0}}\cdot \Delta \lambda_{{\mathrm{TM}}_{0}}-S_{T,{\mathrm{TM}}_{0}}\cdot \Delta \lambda_{{\mathrm{TE}}_{0}}}{\det({\mathrm{SM}}_{T,\mathrm{RI}})}$$

From Equations (7) and (8), it is clear that changes in temperature and RI are calculated using the combined effect of wavelength deviation represented as $\Delta \lambda_{{\mathrm{TE}}_{0}}$ and $\Delta \lambda_{{\mathrm{TM}}_{0}}$ due to ${\mathrm{TE}}_{0}$ and ${\mathrm{TM}}_{0}$ polarizations, respectively, which ensures accurate measurement.

The sensitivity of SOI-based MRR is attributed to the corresponding deviation in the effective refractive index, which is induced by variations in the surrounding temperature and RI. This relationship was previously established in [38].

(9) $${\mathrm{Sensitivity}}_{\mathrm{var},p}=\frac{\delta \lambda_{p}}{\delta \mathrm{var}}=\left(\frac{\delta n_{\mathrm{eff},p}}{\delta \mathrm{var}}\right)\times \left(\frac{\delta \lambda_{p}}{\delta n_{\mathrm{eff},p}}\right)$$

where δvar represents the change in the variable, either RI or temperature; p denotes the polarization, either ${\mathrm{TM}}_{0}$ or ${\mathrm{TE}}_{0}$; $\lambda_{p}$ denotes the resonance wavelength corresponding to polarization state *p*; and $\delta n_{\mathrm{eff},p}$ indicates the change in the effective refractive index for polarization *p*.

The ambient variations change the $n_{\mathrm{eff}}$, and this can be described by

(10) $$\frac{\delta n_{\mathrm{eff},p}}{\delta \mathrm{var}}\left(\lambda \right)=A_{\mathrm{clad},p}\left(\lambda \right)\cdot \frac{\delta n_{\mathrm{clad}}}{\delta \mathrm{var}}\left(\lambda \right)+A_{c,p}\left(\lambda \right)\cdot \frac{\delta n_{c}}{\delta \mathrm{var}}\left(\lambda \right)+A_{\mathrm{buff},p}\left(\lambda \right)\cdot \frac{\delta n_{\mathrm{buff}}}{\delta \mathrm{var}}\left(\lambda \right)$$

Here, $A_{\mathrm{clad},p}$, $A_{c,p}$, and $A_{\mathrm{buff},p}$ represent the confinement factors in the cladding, core, and buffer regions, respectively, for polarization *p*, as illustrated in Figure 12. If the ambient RI changes, no change is induced in ($n_{\mathrm{eff}}$) for the core and buffer layers, as $\frac{\delta n_{c}}{\delta n}\left(\lambda \right)=0$ and $\frac{\delta n_{\mathrm{buff}}}{\delta n}\left(\lambda \right)=0$, respectively. Regarding the effect of temperature changes on the cladding, silicon, and silica layers, it is essential to consider the thermo-optic coefficients of these materials. Given that the thermo-optic coefficients [39,40] for liquid solvents and silicon layers are significantly higher than those for silica, the impact of the silica layer on temperature variation can be disregarded. Consequently, using Equation (6), we derive the following equations:

(11) $$Sensitivity_{n,p}=A_{\mathrm{clad},p}\left(\lambda \right)\cdot \frac{\delta n_{\mathrm{clad}}}{\delta n}\left(\lambda \right)\cdot \frac{\delta \lambda_{p}}{\delta n_{\mathrm{eff},p}}$$

(12) $$Sensitivity_{T,p}=A_{\mathrm{clad},p}\left(\lambda \right)\cdot \frac{\delta n_{\mathrm{clad}}}{\delta T}\left(\lambda \right)+A_{c,p}\left(\lambda \right)\cdot \frac{\delta n_{c}}{\delta T}\left(\lambda \right)\frac{\delta \lambda_{p}}{\delta n_{\mathrm{eff},p}}$$

For the equations presented, $Sensitivity_{n,TM_{0}}$ will be greater than $Sensitivity_{n,TE_{0}}$. This discrepancy arises because $Sensitivity_{n,p}$ is strongly influenced by the confinement factor within the cladding region, and the ${\mathrm{TM}}_{0}$ polarization exhibits greater field intensity within this region. Considering the substantially lower thermo-optic coefficient of air compared to that of silicon [32,41], $Sensitivity_{T,TE_{0}}$ is correspondingly lower in air than in the silicon layer. Additionally, $Sensitivity_{T,TM_{0}}$ remains low in silicon due to the predominance of ${\mathrm{TE}}_{0}$ fields within the silicon core, as opposed to ${\mathrm{TM}}_{0}$ fields, which are more concentrated in the cladding region.

Consequently, the sensitivities to both RI and temperature vary between ${\mathrm{TE}}_{0}$ and ${\mathrm{TM}}_{0}$ polarizations. The incorporation of angular gratings facilitates the simultaneous measurement of the RI and temperature, enabling a broader range of temperature assessment. Utilizing Equations (5) and (6) and incorporating sensitivity values corresponding to specific polarizations, we formulate the following equation:

(13) $$\left[\begin{array}{c} \Delta T\\ \Delta n \end{array}\right]={\left[\begin{array}{cc} 13.33\;\mathrm{nm}/\mathrm{RIU} & 334\;\mathrm{pm}{/}^{\circ }C\\ 76.66\;\mathrm{nm}/\mathrm{RIU} & 260\;\mathrm{pm}{/}^{\circ }C \end{array}\right]}^{-1}\left[\begin{array}{c} \Delta \lambda_{{\mathrm{TE}}_{0}}\\ \Delta \lambda_{{\mathrm{TM}}_{0}} \end{array}\right]$$

One key parameter for assessing sensor performance is the limit of detection (LOD). In this work, we primarily focus on extending the FSR for temperature measurement while achieving an improved LOD, simultaneously monitoring both the RI and temperature. Using the expression presented in [42], our structure achieves an LOD of approximately $2.99\times 10^{-5}$ for the ${\mathrm{TE}}_{0}$ mode, which is employed for temperature sensing, while the LOD for the ${\mathrm{TM}}_{0}$ mode is $1.14\times 10^{-2}$:

(14) $$\mathrm{LOD}=\frac{\lambda_{\mathrm{res}}}{QS}$$

As shown in Table 1, our structure exhibits a superior LOD and sensitivity for temperature variations, making it particularly suitable for applications where ambient temperature changes are of prime importance.

To assess the viability of simultaneous measurements with our device, we first set the temperature $T_{\mathrm{set}}$ to 27 °C and the refractive index ${\mathrm{RI}}_{\mathrm{set}}$) to 1 (the refractive index of air) in the simulation setup. The corresponding resonant wavelengths, $\lambda_{T,\mathrm{set}}$ and $\lambda_{\mathrm{RI},\mathrm{set}}$, were measured to be 1550.07 nm and 1549.81 nm, respectively. Using Equation (13), we then varied the values of temperature and refractive index (RI) and calculated $\Delta \lambda_{{\mathrm{TE}}_{0}}$ and $\Delta \lambda_{{\mathrm{TM}}_{0}}$ for each variation group with respect to the set conditions. Table 2 presents the measured values of $\Delta \lambda_{{\mathrm{TE}}_{0}}$ and $\Delta \lambda_{{\mathrm{TM}}_{0}}$ for six distinct groups, enabling the determination of changes in temperature (ΔT) and RI (Δn).

## 4. Conclusions

In this study, we introduced a dual-polarization angular-grating microring resonator (DP AG-MRR)-based structure incorporating angular gratings and a directional coupler-like coupling section, designed to extend the measurement range for temperature variations. For the first time, we explored the capability of the angular-grating MRR (AG-MRR) to simultaneously detect changes in temperature and RI across dual polarization modes within its transmission spectra, thereby achieving a comprehensive measurement range for ambient temperature variations. Through parameter optimization, we established a set of resonance pairs with distinct sensing ratios for temperature and RI. The simulated RI sensitivities of 13.33 nm/RIU and 76.66 nm/RIU, along with temperature sensitivities of 334 pm/°C and 260 pm/°C, were obtained by analyzing the resonance shift in the modes in response to RI variations at different temperatures. The simulations yielded an FSR of about 35 nm with a detection limit as low as $2.99\times 10^{-5}$, significantly enhancing the measurement range for temperature while concurrently sensing the ambient RI. Furthermore, we assessed the impact of dimensional deviations in the angular gratings and coupling region parameters, such as the coupling gap $g_{c}$, grating gap $W_{g}$, and duty cycle (D), on the performance of the DP AG-MRR. Given its straightforward configuration and compatibility with various silicon-on-insulator (SOI) devices, this design proves advantageous for applications requiring simultaneous detection with a single measurement including a larger temperature range.

## Figures and Tables

**Table 1:** Comparative analysis with other MRR structures.

References	Structure Type	Parameters	RI Sensitivities (nm/RIU)	T Sensitivities (pm/°C)	Range RI; T (nm)	LOD (/RIU); (/°C)
[19]	AG-MRR	Single	95.27 pm/%	NA ^1^	50.2 (RI)	0.0032
[35]	DP-MRR	Dual	104; 319	78.7; 34.1	Limited by FSR	$3.8\times 10^{-4}$; 0.5
[36]	SWGMRR	Single	366	NA ^1^	Limited by FSR	NM ^2^
[37]	AG-SWGMRR	Single	672.8	NA ^1^	NM ^2^	$6.69\times 10^{-5}$
This Work	DP-AGMRR	Dual	76.66; 13.33	260; 334	7.6; 35 (FSR Free for T)	$1.14\times 10^{-2}$; $2.99\times 10^{-5}$

^1^ Not applicable. ^2^ Not mentioned.

**Table 2:** Measurement of temperature and RI simultaneously.

$\Delta T_{\mathrm{base}}\;{(}^{\circ }C)$	$\Delta n_{\mathrm{base}}$	$\Delta \lambda_{TE_{0}}\;\left(\mathrm{nm}\right)$	$\Delta \lambda_{TM_{0}}\;\left(\mathrm{nm}\right)$	$\Delta n_{\mathrm{cal}}$	$\Delta T_{\mathrm{cal}}\;{(}^{\circ }C)$
0	0.03	0.4	2.3	0.030	0.001
3	0.01	1.51	3.24	0.010	3.33
5	0	1.4	1.6	0.003	5.55
8	0.02	3.5	7.0	0.028	8.05
12	0.015	4.6	6.9	0.025	11.98
13	0.025	5.33	9.65	0.044	12.87

## Data Availability

All original findings presented in this study are contained within the article.

## Abbreviations

The following abbreviations are used in this manuscript:

SOI	Silicon-On-Insulator;
TE	Transverse Electric;
TM	Transverse Magnetic;
RI	Refractive Index;
SPR	Surface Plasmon Resonance;
MRR	Microring Resonator;
FSR	Free Spectral Range;
MCRW	Metal-Clad Ridge Waveguide;
DP AG-MRR	Dual-Polarization Angular-Grating microring resonator;
ICP-RIE	Inductively Coupled Plasma Reactive Ion Etching;
CW	Continuous Wave;
PML	Perfectly Matched Layer;
ER	Extinction Ratio;
QF	Quality Factor;
LOD	Limit of Detection.
